# Supplementary material for: Different Features of Interleukin-37 and Interleukin-18 as Disease Activity Markers of Adult-Onset Still’s Disease
Source: J Clin Med. 2021 Feb 26;10(5):910. doi: 10.3390/jcm10050910 (PMC7956170; doi:10.3390/jcm10050910)
Supplement: Supplementary file 1 [file jcm-10-00910-s001.pdf]

Supplementary Material

Table S1. Yamaguchi criteria for the diagnosis of adult-onset Still's disease

Major criteria	Fever ≥ 39ºC lasting ≥ 1 week
	Arthralgia or arthritis lasting ≥ 2 weeks
	Nonpruritic salmon-colored rash (usually over trunk or extremities while febrile)
	Leukocytosis (≥ 10,000/mm^3) with granulocyte predominance (≥ 80%)
Minor criteria	Sore throat
	Lymphadenopathy
	Hepatomegaly or splenomegaly
	Abnormal liver function test
	Negative tests for antinuclear antibody and rheumatoid factor
Exclusion criteria	Infection
	Malignancy
	Other rheumatic disease
At least 5 criteria, including 2 major criteria and no exclusion criteria, are required for diagnosis.	

Table S2. Comparison of clinical characteristics between the IL-37 and IL-18 dominant groups in patients with adult-onset Still's disease[a]

	IL-37 dominant group (N=10)	IL-18 dominant group (N=10)	p
Age	49 (39 – 58)	44 (37 – 52)	0.63
Female, n (%)	9 (90.0)	8 (80.0)	1.00
Disease duration (months)	41 (18 – 88)	50 (19 – 122)	0.74
Number of Yamaguchi criteria met, at diagnosis	7 (6 – 7)	7 (6 – 7)	0.87
AOSD disease course, n (%)			0.73
Monocyclic systemic	3 (30.0)	4 (40.0)	
Polycyclic systemic	3 (30.0)	4 (40.0)	
Chronic articular	4 (40.0)	2 (20.0)	
Number of disease flares (per year)	1 (1 – 2.5)	3 (1 – 4.5)	0.50
Disease activity parameters at high disease activity status			
Modified Pouchot score	7 (6 – 7)	6 (6 – 7)	0.97
Fever, n (%)	9 (90)	9 (90)	1.00
Evanescent rash, n (%)	9 (90)	7 (70)	0.58
Sore throat, n (%)	9 (90)	5 (50)	0.14
Arthritis, n (%)	9 (90)	8 (80)	1.00
Myalgia, n (%)	8 (80)	9 (90)	1.00
Pleuritis, n (%)	0 (0)	3 (30)	0.21
Pericarditis, n (%)	0 (0)	1 (10)	1.00
Pneumonitis, n (%)	3 (30)	3 (30)	1.00
Lymphadenopathy, n (%)	2 (20)	2 (20)	1.00
Hepatomegaly or abnormal LFT[b], n (%)	6 (60)	8 (80)	0.63
Hepatomegaly	1 (10)	1 (10)	1.00
Abnormal LFT[b]	5 (50)	8 (80)	0.35
WBC > 15,000 (10^9/L), n (%)	6 (60)	3 (30)	0.37
Ferritin > 3,000 (ng/mL), n (%)	2 (20)	7 (70)	0.07

Laboratory findings			
WBC (10^9/L)	16.4 (8.4 – 24.0)	10.8 (6.3 – 17.0)	0.25
ESR (mm/hr)	102.5 (62.0 – 115.0)	73.0 (36.0 – 83.0)	0.09
CRP (mg/dL)	8.8 (5.3 – 15.0)	4.1 (2.8 – 7.6)	0.11
AST (U/L)	30.5 (22.0 – 40.0)	42.0 (29.0 – 69.0)	0.17
ALT (U/L)	23.5 (12.0 – 49.0)	45.0 (17.0 – 90.0)	0.12
LDH (U/L)	302.0 (246.0 – 354.0)	492.0 (355.0 – 522.0)	0.01
Ferritin (ng/mL)	2069.5 (727.0 – 2628.0)	5246.5 (2268.0 – 11254.0)	0.11
Medications used at high disease activity status			
Corticosteroids, n (%)	8 (80.0)	7 (70.0)	1.00
Methotrexate, n (%)	7 (70.0)	3 (30.0)	0.18
Leflunomide, n (%)	0 (0)	3 (30.0)	0.14
Cyclosporin A, n (%)	1 (10.0)	1 (10.0)	1.00
Interval between high and low disease activity status (months)	8 (4 – 9)	4 (3 – 6)	0.06
Patients with re-flare within 1 year after low disease activity status, n (%)	2 (33.3)	3 (50.0)	1.00
Duration until re-flare (months)	3.0 (2.2 – 11.5)	4.8 (4.1 – 5.5)	0.77

IL, interleukin; AOSD, adult-onset Still's disease; LFT, liver function test; WBC, white blood cells; ESR, erythrocyte sedimentation rate; CRP, C-reactive protein; AST, aspartate aminotransferase; ALT, alanine aminotransferase; LDH, lactate dehydrogenase.

[a] Values are median (Q1-Q3) unless otherwise indicated.

[b] Defined as abnormal value of AST, ALT, or alkaline phosphatase
